# Diagnosing Malaria Patients with *Plasmodium falciparum* and *vivax* Using Deep Learning for Thick Smear Images

**DOI:** 10.3390/diagnostics11111994

**Published:** 2021-10-27

**Authors:** Yasmin M. Kassim, Feng Yang, Hang Yu, Richard J. Maude, Stefan Jaeger

**Affiliations:** 1 National Library of Medicine, National Institutes of Health, Bethesda, MD 20894, USA; feng.yang2@nih.gov (F.Y.); hang.yu@nih.gov (H.Y.); 2 Mahidol-Oxford Tropical Medicine Research Unit, Faculty of Tropical Medicine, Mahidol University, Bangkok 10400, Thailand; richard@tropmedres.ac; 3 Centre for Tropical Medicine and Global Health, Nuffield Department of Medicine, University of Oxford, Oxford OX3 7LG, UK; 4 Harvard TH Chan School of Public Health, Harvard University, Boston, MA 02115, USA

**Keywords:** malaria, computer-aided diagnosis, biomedical image analysis, deep learning, ResNet50, Mask R-CNN, Plasmodium parasite, *Plasmodium falciparum*, *Plasmodium vivax*

## Abstract

We propose a new framework, PlasmodiumVF-Net, to analyze thick smear microscopy images for a malaria diagnosis on both image and patient-level. Our framework detects whether a patient is infected, and in case of a malarial infection, reports whether the patient is infected by *Plasmodium falciparum* or *Plasmodium vivax*. PlasmodiumVF-Net first detects candidates for Plasmodium parasites using a Mask Regional-Convolutional Neural Network (Mask R-CNN), filters out false positives using a ResNet50 classifier, and then follows a new approach to recognize parasite species based on a score obtained from the number of detected patches and their aggregated probabilities for all of the patient images. Reporting a patient-level decision is highly challenging, and therefore reported less often in the literature, due to the small size of detected parasites, the similarity to staining artifacts, the similarity of species in different development stages, and illumination or color variations on patient-level. We use a manually annotated dataset consisting of 350 patients, with about 6000 images, which we make publicly available together with this manuscript. Our framework achieves an overall accuracy above 90% on image and patient-level.

## 1. Introduction

Malaria is a contagious and potentially deadly disease attributable to Plasmodium (P.) parasites carried and transmitted to humans through mosquito bites. According to the World Health Organization (WHO) [1], there were approximately 229 million cases in 2019, with more than 400,000 worldwide death cases. Most of those cases are in the African region, and children, pregnant women, patients with HIV/AIDS, and travelers are the most at-risk groups. The symptoms appear within 15 days after infection, and if not discovered and treated within 24 h, severe illness and serious consequences may occur, including death.

The microscope is the gold standard for a malaria diagnosis [2]. Microscopy is used to identify the infection after a microscopist places a drop of blood on a glass slide, stains it, and checks it for parasites. Malaria primarily spreads in poor African countries that lack equipment, materials, and individuals with sufficient expertise to report a reliable diagnosis [1]. Malaria symptoms overlap with those of other diseases with similar symptoms, which can lead to increased antibiotic and drug resistance when treatments are based on the symptoms alone [3,4]. On the other hand, it is extremely dangerous and may be fatal to leave malaria untreated if a person is actually infected. Automated algorithms using image processing, computer vision, and artificial intelligence are continuously evolving [5,6,7] and can help to alleviate this problem by providing more reliable and standardized decisions, especially in resource-poor regions. Moreover, the algorithms could benefit researchers, allowing them to quickly evaluate their experiments without expensive lab equipment.

For malaria screening, computer scientists are developing algorithms for thin and thick smears [2,8,9,10,11,12]. Blood smears are used to determine whether a person is infected, report parasite density, and identify parasite species [13]. Thin smears are normally used for species identification and thick smears are often used for a first decision as to whether a patient has malaria because thin smears may not be adequate to identify parasites in individuals with low parasitemia, while thick blood smears are often used to inspect a larger volume of blood [8,14,15,16]. We think that it would make diagnostics more efficient if malaria species could be automatically detected on thick smears as well.

Our proposed framework analyzes thick smear images for malaria diagnosis. Detecting and classifying parasites in thick blood smears is a challenging process. Parasites are noise-like structures and extremely small in high-resolution images that suffer from staining artifacts and illumination variations. In our dataset, the parasite radius can only be 2 pixels in an image of 3k by 4k pixel resolution.

Reporting a patient-level decision is even more challenging because any detection algorithm likely produces false positives in an image when the patient data consists of several images. Our proposed framework tackles those challenges and adopts specific criteria to report the patient-level decision.

We structured our paper as follows: Section 1 introduces the problem, presents a literature review for algorithms that process thick smear images in malaria microscopy, and discusses our contribution. Section 2 describes the datasets that we use in our experiments and introduces the methodology. Section 3 presents the experimental network settings and discusses the quantitative performance. Finally, Section 4 concludes the paper with the main result.

### 1.1. Literature Review

Malaria is caused by Plasmodium parasites. There are five known Plasmodium parasite species causing malaria: *P. falciparum*, *P. vivax*, *P. malariae*, *P. ovale*, and *P. knowlesi*. According to the WHO [1], *P. falciparum* and *P. vivax* are the most deadly parasites and pose the greatest risk. Most of the computational analysis algorithms presented in the literature perform a patch-level evaluation for identifying *P. falciparum* parasites. To the best of our knowledge, our work is the first to identify patients with *P. falciparum* and *P.vivax* parasites in a large dataset of thick smear images and that provides an image and patient-level decision on the infection. The literature review is ordered from older to more recent papers found in the literature.

In 2011, Kaewkamnerd et al. [17] analyzed the V-value histogram of the hue, saturation, and value (HSV) image and extracted white blood cells (WBCs) and parasites using adaptive thresholding. They identified two species (*P. falciparum* vs. *P. vivax*) and classified them based on size. This approach is not reliable because the size is not a robust distinguishing feature and they only processed 20 images with 60% overall accuracy.

In 2011, Elter et al. [18] detected Plasmodium parasites by looking for objects containing chromatin and filtered out the non-parasite objects based on shape and intensity. Then, they used a support vector machine (SVM) with a set of features to identify parasites. The algorithm is applied on 256 images and only to *P. falciparum* with a patch-level evaluation.

In 2013, Purnama et al. [19] developed a three-stage algorithm beginning with preprocessing, feature extraction based on color space histogram, and genetic programming for classification. They ran their algorithm on 180 image patches to classify different Plasmodium species. There is no detection step, and the evaluation is on patch level.

In 2014, Quinn et al. [20] collected overlapping patches and considered a patch as positive if it had a parasite in the center. They collected features derived from connected components and from calculating moments of the patches thresholded at multiple levels. Then, they used a randomized trees classifier to classify the patches. They ran the algorithms on 133 patients and 2703 images. The algorithm only evaluated on patch level, and is only applied to a dataset of infected patients. As a result, the authors did not test it on uninfected patients to check if it recognized them as uninfected. Moreover, the algorithm could not recognize different species; it only recognized Plasmodium parasites in general.

In 2015, Chakrabortya et al. [21] detected parasites based on an algorithm consisting of several modules for grayscale conversion, binarization, morphological operation, and color-based discrimination. They only used 75 images for *P. vivax* and the algorithm was not tested on images with *P. falciparum* parasites or on uninfected images. Moreover, the evaluation is on patch level, not on patient level.

In 2015, Delahunt et al. [22] proposed an algorithm that finds candidate objects using a segmentation module and traditional feature engineering with convolutional neural networks. The engineered features include morphological, color, texture, and rectangular Haar features. They trained and tested their algorithm using *P. falciparum* and negative samples. They tested the algorithm on *P.vivax* patients; however, in their discussion section, they state that their results apply only to *P. falciparum*.

In 2016, Rosado et al. [23] developed an algorithm to detect *P. falciparum* trophozoites and white blood cells in Giemsa-stained thick blood smears. They used an SVM classifier and a total of 314 image features extracted for each candidate. The evaluation is only on patch level; they used 6 patients with 194 images, and only addressed *P. falciparum* parasites and WBCs.

In 2017, Dave et al. [24] extracted parasites from thin and thick blood smear images; they discriminated between the two based on histogram type (bimodal vs. unimodal). After recognizing the image type, separate pipeline steps were designed for each type based on color space conversions, adaptive thresholding, and connected components. They processed 30 thin blood smear images, and 87 thick blood smear images, and presented parasite counting results on patch level.

In 2017, Mehanian et al. [25] provided a multi-module processing pipeline consisting of the following modules: (1) a preprocessing module based on a new sample-level global white balance method that pools the pixels from all of the fields of view (FoVs) and computes a global color balance affine transform for each blood sample, (2) an object detection module based on a novel adaptive nonlinear grayscale intensity image, (3) a feature extraction module incorporating CNNs and introducing a new gamma-transforming color augmentation scheme, (4) a CNN classification module, and finally, (5) a disposition module that computed a patient-level diagnosis and quantification. They stated that their pipeline is the first that applies CNN models with sufficient data, 1452 images and 195 patients, and introduced patient-level accuracy. However, they only identified *P. falciparum* parasites.

In 2020, Yang et al. [26] used an intensity-based iterative global minimum screening (IGMS) method for fast and automatic preselection of parasite candidates and a customized CNN model for classification between parasites and non-parasite patches. The IGMS worked well for *P. falciparum*; however, the parameters are optimized to handle only *P. falciparum* parasites. They tested the method on 150 *P. falciparum* patients with 1818 thick smear images with 84,961 cells. Therefore, the algorithm is applicable only to patients infected with *P. falciparum*.

In 2020, Chibuta et al. [27] modified YOLOV3 to handle small object detections. They applied the network to two datasets with 2703 images from 133 individuals infected by *P. falciparum* parasites. The evaluation is done only on patch level.

In 2021, Abdurahman et al. [28] modified YOLOV3 and YOLOV4 to handle small object detections. They tested their modified network on 1182 images from patients infected by *P. falciparum* parasites. They stated that they will handle other species in their future work. The evaluation was done only on patch level.

In 2021, Horning et al. [29] implemented a fully-automated system, named EasyScan GO, to detect malaria parasites and identify parasite species. They stated that a successful distinction between non-falciparum species using only thick films has not yet been achieved and the algorithm still depends on thin films for this task. In our framework, however, we are able to produce patient-level parasite species identification between *P. falciparum* and *P. vivax* based on thick blood smear microscopy images.

### 1.2. Contribution

**Figure 1:**
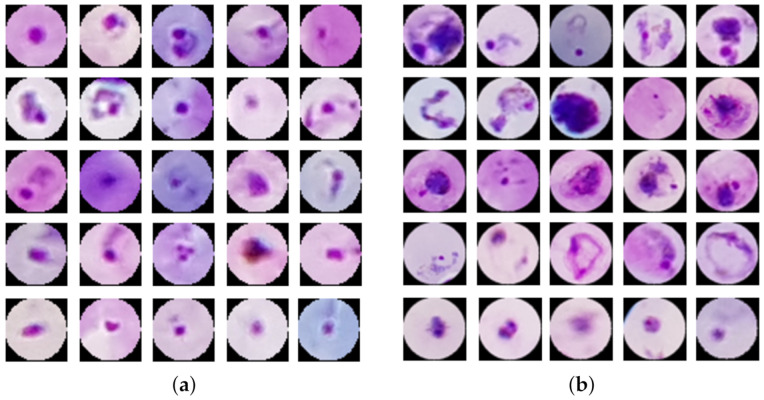
Examples of *P. vivax* and *P. falciparum* parasites. We extract those samples using the gold standard ground truth annotated by an expert reader. We resize the parasites to 44 × 44 for better visualization and consistency. (**a**) *P. falciparum* parasites. (**b**) *P. vivax* parasites.

The literature lacks a complete framework that can both detect whether a patient is uninfected or infected and can recognize the parasite species causing an infection. The methods either detect only one parasite species or classify manually extracted patches of different Plasmodium species. Most of the authors evaluated their work on patch level and did not report the performance of their method on patient level. Plasmodium species differ in size, shape, and morphology; see Figure 1 and Table 1. We assume, realistically, that we do not know beforehand whether a patient is uninfected or infected. Furthermore, if infected, we assume that the system has no prior knowledge of whether the patient is infected by *P. falciparum* or *P. vivax*.

We design the first framework (PlasmodiumVF-Net) that addresses the problem of detecting and classifying parasites and reporting a patient-level decision for thick smear microscopy. In this framework, we determine the parasite species based on the number of detected patches and aggregated probabilities of the predicted patches for all of the patient images. We utilize three datasets to evaluate our framework: a dataset with 150 patients infected by *P. falciparum* parasites, a dataset with 150 patients infected by *P. vivax* parasites, and a dataset with images from 50 uninfected patients. In total, we will make 350 patients and 5972 images open source with the publication of this paper. To the best of our knowledge, this is the first public dataset containing annotated thick smear images for *P. vivax* parasites.

## 2. Materials and Methods

### 2.1. Data Set

We used three datasets of Giemsa-stained thick blood smears that are photographed at Chittagong Medical College Hospital, Bangladesh, through the eyepiece of a microscope with 100× magnification, using a smartphone camera, and manually annotated by an experienced expert. We collected, de-identified, and archived all of the images and their annotations at the National Library of Medicine (IRB#12972).

All images in the datasets are from 350 infected and uninfected patients. The first dataset is the *P. falciparum* dataset which we have already published in [26] under this link: https://data.lhncbc.nlm.nih.gov/public/Malaria/Thick_Smears_150/index.html (last accessed 26 October 2021). The second dataset is the *P. vivax* dataset that we publish with this article under this link: https://data.lhncbc.nlm.nih.gov/public/Malaria/NIH-NLM-ThickBloodSmearsPV/NIH-NLM-ThickBloodSmearsPV.zip (last accessed 26 October 2021). Both of the datasets are acquired from infected patients. The third dataset is from uninfected patients and is also released with this article here: https://data.lhncbc.nlm.nih.gov/public/Malaria/NIH-NLM-ThickBloodSmearsU/NIH-NLM-ThickBloodSmearsU.zip (last accessed 26 October 2021). All images are in RGB color with a resolution of 3024 × 4032 pixels.

**Figure 2:**
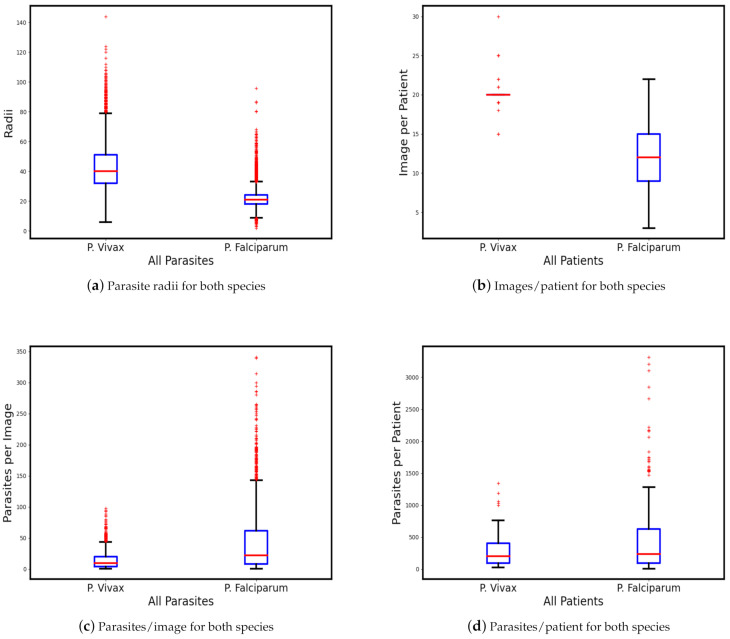
Range comparison of our two parasite datasets in terms of parasite radius (**a**), number of images per patient (**b**), number of parasites per image (**c**), and number of parasites per patient (**d**). The box plots display the boxes’ bottom, median, and top edges for the 25th, 50th, and 75th percentiles, respectively. The outliers are plotted as individual points by a red mark beyond the whiskers.

Dataset statistics: Table 1 lists the main statistical differences between our two datasets for infected patients. The same number of patients has more images for *P. vivax*, and more infected cells for *P. falciparum*. *P. vivax* parasites have a larger radius than *P. falciparum* parasites. To visualize these numbers, we generate four box plots in Figure 2. The box plots display the bottom, median, and top edges of the boxes for the 25th, 50th, and 75th percentiles, respectively. The outliers are plotted as individual points by a red + mark beyond the whiskers. A data point is considered an outlier if its value is 1.5 times higher than the interquartile range from both box edges.

Figure 2a shows the parasite radii for both datasets. For example, there are 560 parasites with a radius higher than 79.5 (top whisker) for the *P. vivax* dataset, while for *P. falciparum*, there are 1358 parasites with a radius higher than 33 (top whisker) and 108 with a radius less than 9 (bottom whisker). Figure 2b displays the number of images per patient for both datasets. *P. vivax* has a more consistent number of images per patient than *P. falciparum*. Figure 2c displays the number of parasites per image, and shows that the *P. falciparum* images have a higher parasite density than the *P. vivax* images. The last subfigure (d) shows a parasite analysis on patient-level.

Figure 1 shows two sets of parasite patches. Set (a) visualizes a sample of *P. falciparum* parasites and set (b) visualizes a sample of *P. vivax* parasites.

### 2.2. Methodology

**Figure 3:**
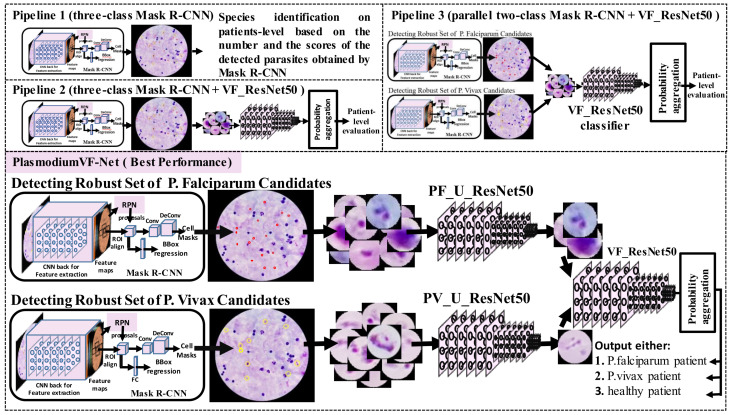
Visualization of our pipelines. The flowchart for PlasmodiumVF-Net is shown in Figure 4.

**Figure 4:**
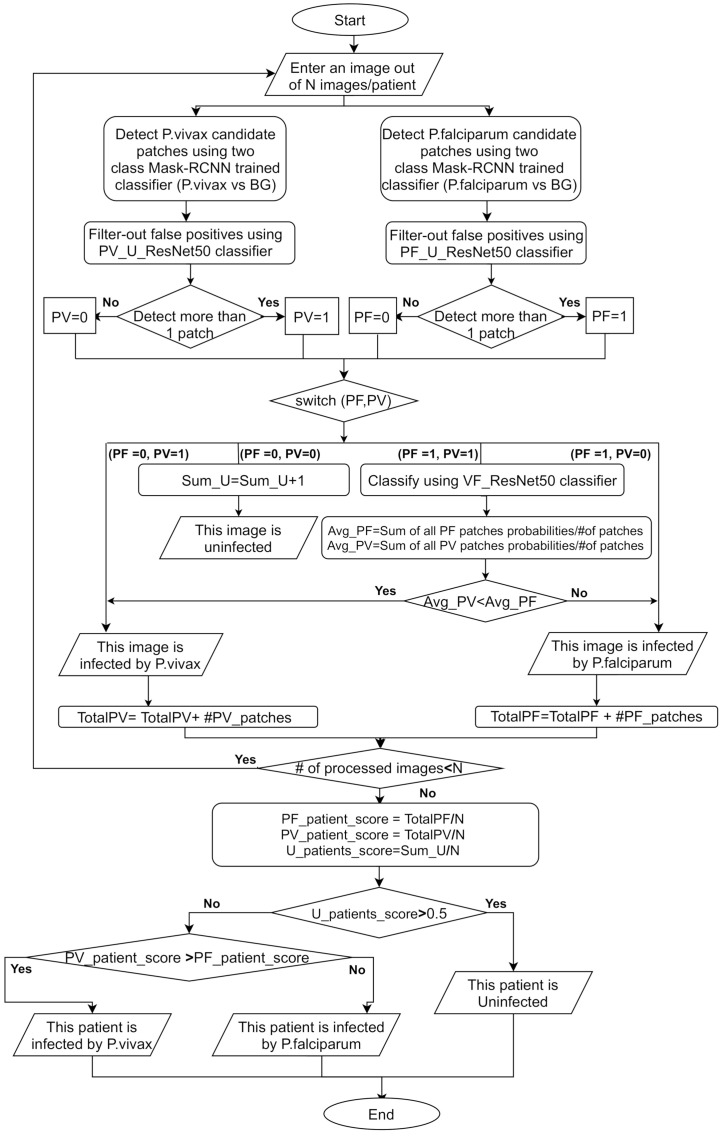
Flowchart for PlasmodiumVF-Net.

We design PlasmodiumVF-Net in stages, and we perform a performance evaluation to validate its effectiveness for each stage. Specifically, we design four pipelines starting from a straightforward model based on Mask R-CNN detection, and add three other classifiers to discriminate between *P. vivax*, *P. falciparum*, and uninfected patients. In this section, we discuss adapting Mask R-CNN for detecting parasites in thick blood smear microscopy. We present a benchmark evaluation for two-class patch classification between *P. vivax* and *P. falciparum*. The benchmark is important to choose the best CNN network classifier for our framework. Finally, we discuss the four pipelines that we develop until we reach our final framework, PlasmodiumVF-Net. Our framework is based on several flags, counters, and scores to compute the image and patient-level decisions. Table 2 shows these variables. The four pipelines are visualized in Figure 3. Figure 4 depicts the flowchart of the complete framework.

#### 2.2.1. Adapting Mask R-CNN for Parasite Detection

Object detection plays a significant role in many applications, ranging from moving object tracking to detecting organs in biomedical images. Recently, several powerful networks for delineating objects have been introduced in the literature, such as Faster R-CNN [30] which has been developed as a successor of two well-known region-based convolutional neural networks, R-CNN [31] and Fast-R-CNN [32]. YOLO (You Only Look Once) [33] is another fast and accurate object detection network that is grid-based rather than region-based. Each of these networks provides a bounding box around the object that includes some background pixels. Instance segmentation is detection that covers object pixels only. Mask R-CNN [34] is one of the state-of-the-art networks in instance segmentation and is extremely powerful for small objects and biomedical applications [35,36,37,38,39,40].

Mask R-CNN consists of several modules: (1) Backbone: We use a residual network with a 50-layer ResNet50 [41] as a convolutional neural network (CNN) for a feature extraction. (2) Region Proposal Network (RPN): to obtain region proposals with different scales and ratios to generate anchors. (3) Multi-class classifier to decide whether each region of interest (ROI) contains an object or not, and a regressor to predict the bounding box coordinates. (4) A fully connected network is added to achieve pixel-level segmentation and generate object masks on pixel level, commonly known as instance segmentation.

We train a Mask R-CNN to detect *P. falciparum* and *P. vivax* patches. Specifically, we perform two experiments: In the first experiment, we train a three-class classifier (*P. falciparum*, *P. vivax*, Background (BG)). The second experiment involves training of two two-class classifiers, *P. falciparum* vs. BG, and *P. vivax* vs. BG. We find using the two-class classifiers to be more effective than the three-class classifier. The evaluation of Mask-R-CNN detection is discussed in Section 3.2.

#### 2.2.2. Patch-Level Two-Class Classification

We produce a benchmark for two-class patch classification (*P. falciparum* vs. *P. vivax*) using four networks: GoogleNet [42], a CNN that is 22 layers deep with an input image size of 224 × 224; SqueezeNet [43], a CNN with 18 layers and with an image input size of 227 × 227; ResNet50 [41], a CNN that is 50 layers deep with an input image size of 224 × 224; and Inceptionv3 [44], which is 48 layers deep with an image input size of 299 × 299. We noticed a degradation in performance when we use deeper networks such as DenseNet201 [45] and InceptionResNetV2 [46] with 201 and 164 layers, respectively. We only include the best results in our benchmark. All of the networks are pre-trained on the ImageNet [47] database to decrease the convergence time and have a rich feature representation as a starting point to learn a new task. The training and testing details are presented in Section 3.1, whereas the results, performance evaluation, and a discussion can be found in Section 3.2.

#### 2.2.3. Proposed PlasmodiumVF-Net Framework

We anticipate a single three-class trained Mask R-CNN model that can detect and classify *P. falciparum* and *P. vivax*, and report image and patient-level decisions about the infection. However, the challenges are the small parasite size, excessive staining artifacts, and microscopy slide variations on patient level.

We create our framework design in stages. Pipeline 1, shown in the upper left part of Figure 3, reports whether the image has *P. falciparum* or *P. vivax* parasites based on the number and the score of the detected parasites using the three-class Mask R-CNN classifier. We find that Mask R-CNN is not robust enough to produce a final patient-level decision; however, it generates an excellent set of parasite candidates. The evaluation and other details are discussed in Section 3.2. For Pipeline 2, shown in Figure 3 underneath Pipeline 1, we add a ResNet50 classifier that discriminates between *P. falciparum* and *P. vivax* patches after the Mask R-CNN detector. We report the image and patient-level decisions based on probability aggregation for these patches. For Pipeline 3, shown in the right corner of Figure 3, we replace the three-class Mask R-CNN classifier with two two-class Mask R-CNN classifiers to enhance the detection. This design enhances the overall accuracy, as reported in Section 3.2. On the other hand, the design’s main drawback is that the ResNet50 classifier that we place after the Mask R-CNN detector is trained only to recognize the patches infected by *P. falciparum* and *P. vivax* and gives an image and patient-level decision based on probability aggregation of those patches. However, the large number of false positives negatively affects the final decisions. To solve this issue, we can either retrain the ResNet50 classifier to recognize three classes, *P. falciparum*, *P. vivax*, and uninfected patches, or add two more classifiers after each Mask R-CNN model to filter out false positives. We choose the second option because we noticed a wide variation between false positives for both *P. falciparum* and *P. vivax*, and we want the classifier to focus on one task for more accurate results. The full framework design, called PlasmodiumVF-Net, is shown in the bottom part of Figure 3.

To clarify the procedure of computing an image and making a patient-level decision based on probability aggregation and parasite count, we design the flowchart in Figure 4, which shows all of the steps of our proposed PlasmodiumVF-Net framework. The flowchart steps are described below, with all of the utilized variables listed in Table 2:

1. Read an image out of N images per patient.
2. Detect in parallel two sets of candidate patches using Mask R-CNN for both *P. falciparum* and *P. vivax* using the two two-class detectors. We apply here the two detectors because we have no prior knowledge about the parasite species causing the infection or whether the patient is uninfected.
3. Filter out false positives using two binary classifiers named PV_U_ResNet50 and PF_U_ResNet50.
4. Set two flags, PV and PF, to indicate whether the framework detects more than one parasite for *P. vivax* and *P. falciparum*, respectively.
5. Based on PV and PF, there are four possibilities:
  (a) If both flags are zero, our proposed PlasmodiumVF-Net reports the image as uninfected and increases the counter, Sum_U, of the number of uninfected images by one.
  (b) When PV = 0 and PF = 1, then PlasmodiumVF-Net reports that the image contains only *P. falciparum* parasites.
  (c) When PV = 1 and PF = 0, then PlasmodiumVF-Net reports that the image contains only *P. vivax* parasites.
  (d) If both flags are one, this means that there are candidate patches for both *P. falciparum* and *P. vivax*. In this case, all of the candidates need to be tested by the VF_ResNet50 classifier. After testing all the patches, the prediction probabilities are aggregated. The averages, represented by Avg_PV and Avg_PF, are computed by dividing the aggregated probabilities by the number of patches. VF_ResNet50 classifies patches as *P. vivax* if they have probabilities of less than 0.5 and as *P. falciparum* if their probabilities are higher than 0.5. Consequently, if Avg_PV is less than Avg_PF, then the image is considered to contain *P. vivax*; otherwise, *P. falciparum*.
6. At this point, we have an image-level decision, and PlasmodiumVF-Net needs to check some parameters and conditions to produce a patient-level decision. TotalPV and TotalPF accumulate the total number of patches when PlasmodiumVF-Net found that the image is infected by *P. vivax* or *P. falciparum*, respectively.
7. If all N images are processed, go to Step 8, otherwise return to Step 1 to process a new image from the current patient.
8. If the PlasmodiumVF-Net found that more than half of the images of the current patient are uninfected based on U_patients_score, which is calculated by dividing the total number of uninfected images by N, then it considers the patient as uninfected; otherwise, it proceeds to the final step.
9. Calculate the PF_patient_score and PV_patient_score by dividing the total number of detected patches, represented by TotalPF and TotalPV, by N. The PlasmodiumVF-Net decides that the patient is infected by *P. falciparum* parasites if the PF_patient_score is higher; otherwise, the patient is considered to be infected by *P. vivax* parasites.

The flowchart steps are repeated for every patient in the dataset to determine the evaluation performance for the whole dataset. This flowchart can give researchers a strategy to determine image and patient-level decisions for different biomedical problems.

## 3. Results and Discussion

### 3.1. Experimental Network Settings

In this section, we explain the cross-validation experiments and review the parameters used to train our networks.

**Mask R-CNN**: We perform a five-fold cross-validation on patient-level. For training the Mask R-CNN, we use the *P. falciparum* and *P. vivax* dataset, each containing 150 patients. We divide each dataset into five sets and train four sets (120 patients) for each experiment and use one set (30 patients) for testing. Out of the 120 patients used for training, 10 patients are used for the validation. For each experiment, the number of images is balanced to obtain unbiased classifiers. For each experiment (fold), the number of images used for training ranges between 1400 and 1485 for *P. falciparum*, and between 2394 and 2420 for *P. vivax*.

Although the number of *P. vivax* images is higher than the number of *P. falciparum* images, our *P. falciparum* patients have a higher average infection rate, and therefore provide more patches for training. We train Mask R-CNN with a momentum weight of 0.9, a learning rate of 0.001, and 40 epochs. The intersection over union (IoU) for positive anchors (proposals) is greater than 0.7 and less than 0.3 for negative anchors. The network’s weights are initialized with transfer learning of ImageNet weights. Online augmentation (flipping, affine transformation, and Gaussian blur) is used to augment and increase patches for the training stage. Performance evaluation is shown in Table 3 and discussed in Section 3.2.

**Figure 5:**
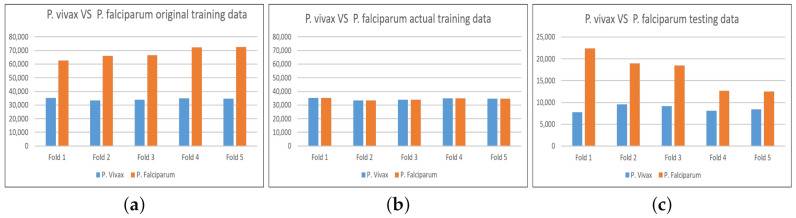
Training and testing statistics for all CNN networks described in Section “Experimental network settings” that classify patches as either *P. falciparum* or *P. vivax* with five-fold cross-validation on patient-level. Subfigure (**a**) shows the statistics for the training data, subfigure (**b**) shows the actual data that has been used for training after balancing the number of *P. falciparum* and *P.vivax* parasites in each fold, and subfigure (**c**) shows the statistics for the testing data.

**CNN network benchmark and VF_ResNet50**: This benchmark, see Table 4, is used to choose the best CNN classifier that can classify *P. falciparum* and *P. vivax* patches. All of the networks (GoogleNet, SqueezeNet, ResNet50, and Inceptionv3) are trained based on patches extracted from the input images using parasite annotations. The training follows the five-fold cross-validation scheme explained earlier in this section. Figure 5 displays three distribution graphs for training and testing of the five folds. Graph (a) shows that the original training data is unbalanced, graph (b) shows the data after we balanced it for each fold by removing patches from the P.falciparum dataset, and graph (c) shows the number of patches for testing. For all of the CNN networks, we use transfer learning based on the pre-trained network of ImageNet as a starting point to take advantage of the early layers with rich low-level features. It is also faster to converge than to learn the network from scratch. To do this, we need to replace the last two layers responsible for class probabilities and computing loss. We retrain and fine-tune the networks to learn a new task based on the new dataset. In addition, we perform an online augmentation using scaling, translation, and reflection. We train the networks for 15 epochs with an initial learning rate of 0.0003. The benchmark shows that ResNet50 is the best; we call this trained model VF_ResNet50, as in Table 2.

**PF_U_ResNet50 and PV_U_ResNet50**: We utilize ResNet50 to train two new classifiers to filter out false positives resulting from the Mask R-CNN detection. In these experiments, we need parasite patches and uninfected patches. We collect all of the false positives from the Mask R-CNN detection to gather uninfected patches rather than collect random uninfected patches that may not feature a stain. Mask R-CNN’s false positives are excellent candidates with staining colors that confuse the Mask R-CNN since most of them are noise or staining artifacts. The two classifiers (PF_U_ResNet50 and PV_U_ResNet50) need to learn that those patches are false positives and need to be eliminated. The training also follows the same patient-level cross-validation discussed above.

### 3.2. Quantitative Performance Evaluation and Discussion

This section presents the performance evaluation for each pipeline.

**(A) Pipeline 1:** We begin our experiments with Pipeline 1 based on Mask R-CNN only. We expected a Mask R-CNN model that can detect whether an image is infected or uninfected and differentiate between *P. vivax* and *P. falciparum* if the image contains parasites; however, that was not the case after a vast number of trials and parameter tuning. Nevertheless, the model produces an excellent set of parasite candidates. We perform two experiments: First, we train a three-class classifier to detect *P. vivax*, *P. falciparum*, and Background (BG). Second, we train two two-class classifiers; one classifier detects *P. vivax* vs. BG and the other one detects *P. falciparum* vs. BG. We found that training two two-class classifiers is more efficient and produces a better performance than a single three-class classifier.

**Figure 6:**
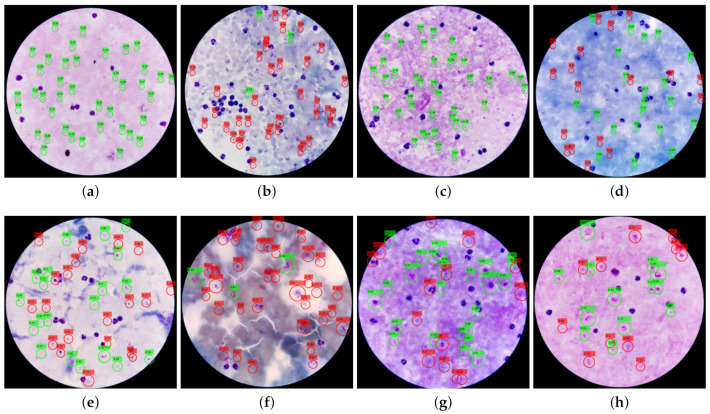
Mask R-CNN parasite detection in eight different patients—four images from different patients (top row) infected with *P. falciparum* and another four from different patients (bottom row) infected with *P. vivax*. The figure shows how the images vary in color and infection rate. Green circles are true positives, while red circles are false positives. We draw the circles larger than the actual parasites for better visualization. Rec and Pre mean recall and precision. (**a**) *P. falciparum* parasite detection with Pre = 100% and Rec = 50%. (**b**) *P. falciparum* parasite detection with Pre = 10% and Rec = 100%. (**c**) *P. falciparum* parasite detection with Pre = 100% and Rec = 10%. (**d**) *P. falciparum* parasite detection with Pre = 50% and Rec = 50%. (**e**) *P. vivax* parasite detection with Pre = 50% and Rec = 80%. (**f**) *P. vivax* parasite detection with Pre = 10% and Rec = 80%. (**g**) *P. vivax* parasite detection with Pre = 60% and Rec = 90%. (**h**) *P. vivax* parasite detection with Pre = 50% and Rec = 80%.

We show the Mask R-CNN detection results in Table 3. The performance evaluation for Mask R-CNN detection results follows the steps discussed in the evaluation section of Kassim et al. [12]. It is noticeable that we achieve a high sensitivity that reaches about 94% for *P. vivax* and 88% for *P. falciparum* using the two-class classifiers. However, Mask R-CNN produces many false positives. These false positives are regions with an appearance similar to parasites due to staining artifacts (non-parasite BG components absorbing the stain), non-uniform illumination, and contrast variations. See Figure 6 for a visualization of the detection results. Green circles represent true positives and red circles represent false positives. The first row of Figure 6 shows *P. falciparum*, while the second row shows *P. vivax*, for different patients. The figure illustrates the color, texture, and illumination variations on patient-level. Moreover, the density of parasites (infection rate) detected by Mask-RCNN differs. For example, subfigure (a) has no false positives and several accurate detections. However, the recall is only 0.5, whereas subfigure (b) has a recall of one with only three parasites and the remaining detections being false positives.

For Pipeline 1 with Mask R-CNN only, we only count the number of detections and compute the image and patient-level evaluations for species identification by aggregating this count. In addition, we consider the highest aggregated probability score if the number of detected patches in an image or patient are the same for the two species. The performance is low, 68.4% on image level and 78.7% on patient level, as shown in Table 5a,b, respectively.

**(B) Pipeline 2:** We add a classifier to classify all of the patches resulting from Mask R-CNN detection as either *P. falciparum* or *P. vivax*. To do so, we choose the best classifier based on our benchmark, which is ResNet50, aggregate all of the probabilities resulting from ResNet50, and average them to compute image and patient-level decisions. Pipeline 2 increases the performance to 77.8% on image level and to 83% on patient level, see Table 5c,d.

**(C) Pipeline 3:** We replace the three-class Mask R-CNN detector with two parallel binary detectors to strengthen the detection part and let each detector focus on one type of parasite, *P. falciparum* or *P. vivax*. This increases the overall accuracy to 83.5% on image level and to 91% on patient level, see Table 5e,f.

**(D) PlasmodiumVF-Net:** After strengthening the detection part to produce the best set of candidates, many false positives still affect the final decision because ResNet50 is only trained to classify patches as *P. falciparum* or *P. vivax*. In other words, if the patch is not a parasite, the classifier produces an arbitrary probability score for this patch that affects the overall image and patient-level decision. For this reason, we filter out the false positives by adding one classifier after each Mask R-CNN detector. This process increases the overall accuracy on image and patient level to 90.8% and 96.7%, respectively, see Table 5g,h.

**Figure 7:**
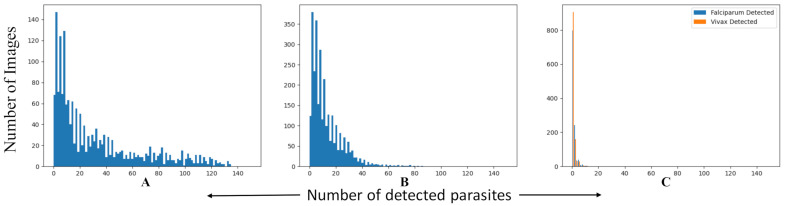
This figure compares the response of PlasmodiumVF-Net for the *P. falciparum*, *P. vivax*, and uninfected patient datasets. (**A**) Subfigure A shows a histogram of parasite detection on image level for *P. falciparum*, (**B**) Subfigure B shows a histogram of parasite detection on image level for *P. vivax*, and (**C**) Subfigure C shows a histogram of both *P. falciparum* and *P. vivax* parasite detection on image level for uninfected patients. From the histograms of Subfigure A and B, it is noticeable that our framework is responding well and identifies the existing parasites in the infected patients, while in Subfigure C, PlasmodiumVF-Net detects only a small number of false-positive parasites for uninfected patients, which shows the effectiveness of our proposed pipeline.

By adding the additional two classifiers, PlasmodiumVF-Net can now decide whether the image or patient is uninfected or infected. This decision is made after filtering the false positive patches. If all of the patches are filtered out, then the image is considered to be uninfected, meaning it contains no parasites. However, this condition is too stringent. The pipeline may still detect one or two parasites even for an uninfected image. We therefore test our pipeline for an additional 50 uninfected patients, with 1141 images that have not been used in the training process. We re-evaluate the three datasets together, and we consider the image to be uninfected when it has less than two parasites (either no parasite or only one parasite); Figure 7 demonstrates why we choose this threshold. Table 6 presents the results. The overall accuracy, ratio of the sum of diagonal values over the total number of samples, is 83.9% on image level and 92.3% on patient level. It is also noticeable that there are more *P. falciparum* images being classified as uninfected compared to *P. vivax* images. *P. falciparum* parasites are harder to detect than *P. vivax* due to their smaller size and similarity to uninfected regions. Figure 7 shows a histogram of PlasmodiumVF-Net responses on patch level. The *y*-coordinate represents the number of images with x parasites as specified by the *x*-coordinate. PlasmodiumVF-Net responds strongly when detecting parasites in infected patients; Figure 7A,B show the histogram of the detected patches, while it shows a faint response, see Figure 7C, when tested on uninfected patients. This confirms the effectiveness of our framework. For normal patients, histogram bin 0 and 1 are high (more than 900 entries), meaning for most of the 1141 images PlasmodiumVF-Net generates either zero or only one false positive parasite. This is the reason why we choose two parasites as our threshold to determine whether the image is taken from an uninfected patient or not.

The processing time for one image is around 6–8 s, which includes making the final decision on whether the image is infected and detecting potential parasites. The processing time for one patient is proportional to the number of images acquired for the patient.

We designed our framework so that it can report whether a patient is uninfected or infected and whether an infection is caused by either *P. falciparum* or *P. vivax*. In the rare case of a mixed infection, our system would make a binary decision and settle for either *P. falciparum* or *P. vivax*, depending on the frequency of each species and probability scores. This is a limitation, which we could easily amend; however, we decided in favor of a binary output for the infected patients because our training and test data contained no mixed infections.

## 4. Conclusions

In this work, we propose four pipelines to compute image- and patient-level infection decisions for Plasmodium parasites. We process challenging thick smear microscopic images from patients infected by *P. falciparum* and *P. vivax* parasites, and from uninfected patients. Our proposed framework, named PlasmodiumVF-Net, reports an infected patient based on patch-level probability aggregation and parasite counting. The framework reaches around 92% overall accuracy on patient level when tested on 350 patients with 5972 images. For future research, we are interested in testing the framework with data from other malaria-endemic regions to analyze whether the framework can handle images and patients from different sources, and in integrating the framework into a smartphone application, such as NLM Malaria Screener [26,48].

## Figures and Tables

**Table 1:** Comparison of two infected datasets: *P. falciparum* and *P. vivax*.

	*P. vivax*	*P. falciparum*
Number of patients	150	150
Number of images	3013	1818
Number of parasites	43,042	84,961
Parasite radius range	6–144	2–96
Average parasite radius	42	22
Number of parasites per image	1–98	1–341
Average number of parasites per image	14	47
Number of images per patient	15–30	3–22
Average number of images per patient	20	12
Number of parasites per patient	24–1345	8–3130
Average number of parasites per patient	287	522

**Table 2:** The definitions of all variables used in this paper.

Variable	Definition
PV_U_ResNet50	ResNet50 classifier is trained to classify patches as either *P. vivax* or uninfected
PF_U_ResNet50	ResNet50 classifier is trained to classify patches as either *P. falciparum* or uninfected
PV	This flag is set if more than one *P. vivax* parasite is still detected after all false positives are filtered out by the PV_U_ResNet50 classifier
PF	This flag is set if more than one *P. falciparum* parasite is still detected after all false positives are filtered out by the PF_U_ResNet50 classifier
VF_ResNet50	ResNet50 classifier is trained to classify patches as either *P. falciparum* or *P. vivax*
Avg_PV	Sum of all probabilities for detected *P. vivax* patches divided by the number of patches detected for a single image
Avg_PF	Sum of all probabilities for detected *P. falciparum* patches divided by the number of patches detected for a single image
TotalPV	Total number of detected *P. vivax* patches for all images of a single patient
TotalPF	Total number of detected *P. falciparum* patches for all images of a single patient
Sum_U	Total number of uninfected images
U_patients_score	Total number of uninfected images divided by the total number of images for a single patient
PV_patient_score	TotalPV/number of images for a single patient
PF_patient_score	TotalPF/number of images for a single patient

**Table 3:** Performance evaluation of Mask R-CNN for detecting *P. vivax* and *P. falciparum* parasites in thick smear microscopy images.

	*P. vivax*		*P. falciparum*	
	**Detection Rate Using** **a Three-Class** **Classifier**	**Detection Rate Using** **a Two-Class** **Classifier**	**Detection Rate Using** **a Three-Class** **Classifier**	**Detection Rate Using** **a Two-Class** **Classifier**
Fold1	85.58	92.94	61.34	83.76
Fold2	82.04	90.60	60.77	86.83
Fold3	88.41	96.70	67.41	87.45
Fold4	89.81	96.22	73.15	90.02
Fold5	88.93	93.68	69.89	90.87
**Avg.**	**86.95**	**94.03**	**66.51**	**87.79**

**Table 4:** Confusion matrices for patch-based two-class classification, *P. falciparum* vs. *P. vivax*, using different networks. Each matrix is the summation of five-fold cross-validation on patient-level. Gray represents target values, whereas yellow represents predicted values. The reported accuracy, written in each table’s caption, is the ratio of the sum of diagonal values over the total number of samples.

(a) GoogleNet Classification Experiments with Average Accuracy Equal to 99.15% [42].		
	*P. falciparum*	*P. vivax*
*P. falciparum*	84,961	1087
*P. vivax*	0	41,955
**(b) SqueezeNet Classification Experiments with Average Accuracy Equal to 99.28% [43].**		
	*P. falciparum*	*P. vivax*
*P. falciparum*	84,961	912
*P. vivax*	0	42,130
**(c) ResNet50 Classification Experiments with Average Accuracy Equal to 99.98% [41].**		
	*P. falciparum*	*P. vivax*
*P. falciparum*	84,961	19
*P. vivax*	0	43,023
**(d) InceptionV3 Classification Experiments with Average Accuracy Equal to 96.76% [44].**		
	*P. falciparum*	*P. vivax*
*P. falciparum*	84,961	4141
*P. vivax*	0	38,901

**Table 5:** Confusion matrices for image and patient-level of our four pipelines using the *P. falciparum* and the *P. vivax* dataset. Each matrix is the summation of five-fold cross-validation on patient-level. Gray represents actual values, whereas yellow represents predicted values. The accuracy for each confusion matrix is mentioned in the caption of each subfigure, measured as the ratio between the sum of the diagonal elements over the total number of images (on image level) or patients (on patient level).

(a) Pipeline 1, Image-Level Identification Results with Accuracy = 68.4%		
	*P. falciparum*	*P. vivax*
*P. falciparum*	1245	952
*P. vivax*	573	2061
Sum of images	1818	3013
**(b) Pipeline 1, Patient-Level Identification Results with Accuracy = 78.7%**		
	*P. falciparum*	*P. vivax*
*P. falciparum*	131	45
*P. vivax*	19	105
Sum of patients	150	150
**(c) Pipeline 2, Image-Level Identification Results with Accuracy = 77.8%**		
	*P. falciparum*	*P. vivax*
*P. falciparum*	1700	955
*P. vivax*	118	2058
Sum of images	1818	3013
**(d) Pipeline 2, Patient-Level Identification Results with Accuracy = 83%**		
	*P. falciparum*	*P. vivax*
*P. falciparum*	141	42
*P. vivax*	9	108
Sum of patients	150	150
**(e) Pipeline 3, Image-Level Identification Results with Accuracy = 83.5%**		
	*P. falciparum*	*P. vivax*
*P. falciparum*	1675	653
*P. vivax*	143	2360
Sum of images	1818	3013
**(f) Pipeline 3, Patient-Level Identification Results with Accuracy = 91%**		
	*P. falciparum*	*P. vivax*
*P. falciparum*	148	25
*P. vivax*	2	125
Sum of patients	150	150
**(g) PlasmodiumVF-Net, Image-Level Identification Results**		
**with Accuracy = 90.8%**		
	*P. falciparum*	*P. vivax*
*P. falciparum*	1756	375
*P. vivax*	52	2630
Sum of images	1808	3005
**(h) PlasmodiumVF-Net, Patient-Level Identification Results**		
with **Accuracy = 96.7%**		
	*P. falciparum*	*P. vivax*
*P. falciparum*	148	8
*P. vivax*	2	142
Sum of patients	150	150

**Table 6:** Confusion matrices for image-level and patient-level three-class classification for PlasmodiumVF-Net. Each matrix is the summation of five-fold cross-validation on patient level. Gray represents actual values, whereas yellow represents predicted values. The accuracy is reported in bold in the caption of each table.

(a) Summation of Confusion Matrices for Five-Fold Cross-Validation of			
PlasmodiumVF-Net with Average Accuracy Equal to 83.9% on Image Level			
	*P. falciparum*	*P. vivax*	Uninfected
*P. falciparum*	1714	337	317
*P. vivax*	36	2537	66
Uninfected	68	139	758
Sum of Images	1818	3013	1141
**(b) Summation of Confusion Matrices for Five-Fold Cross-Validation of**			
**PlasmodiumVF-Net with Average Accuracy Equal to 92.3% on Patient Level**			
	*P. falciparum*	*P. vivax*	Uninfected
*P. falciparum*	145	8	12
*P. vivax*	2	141	**1**
Uninfected	3	1	37
Sum of Patients	150	150	50

## Data Availability

The data used in this research is available via the following links (last accessed 26 October 2021): 1. https://data.lhncbc.nlm.nih.gov/public/Malaria/Thick_Smears_150/index.html. 2. https://data.lhncbc.nlm.nih.gov/public/Malaria/NIH-NLM-ThickBloodSmearsPV/NIH-NLM-ThickBloodSmearsPV.zip. 3. https://data.lhncbc.nlm.nih.gov/public/Malaria/NIH-NLM-ThickBloodSmearsU/NIH-NLM-ThickBloodSmearsU.zip.
